# Leading the Game, Losing the Competition: Identifying Leaders and Followers in a Repeated Game

**DOI:** 10.1371/journal.pone.0150398

**Published:** 2016-03-11

**Authors:** Knut Lehre Seip, Øyvind Grøn

**Affiliations:** Faculty of technology, art and design, Oslo and Akershus University College for Applied Sciences, Oslo, Norway; Peking University, CHINA

## Abstract

We explore a new method for identifying leaders and followers, LF, in repeated games by analyzing an experimental, repeated (50 rounds) game where Row player shifts the payoff between small and large values–a type of “investor” and Column player determines who gets the payoff–a type of “manager”. We found that i) the Investor (Row) most often is a leading player and the manager (Column) a follower. The longer the Investor leads the game, the higher is both player’s payoff. Surprisingly however, it is always the Manager that achieves the largest payoff. ii) The game has an efficient cooperative strategy where the players alternate in receiving a high payoff, but the players never identify, or accept, that strategy. iii) Under the assumption that the information used by the players is closely associated with the leader- follower sequence, and that information is available before the player’s decisions are made, the players switched LF- strategy primarily as a function of information on the Investor’s investment and moves and secondly as a function of the Manager’s payoff.

## Introduction

When two players have conflicting preferences, one of the players has to forego her or his preferred choice, and there is a cost of consensus. Who bears this cost? In principle, and in the game discussed in this study, the cost of consensus can be equally shared between the two players. In practice, however, one player (the leader) may exert a disproportional influence on how the game proceeds, whereas the other (the follower) has less control and tends to conform to the preferences of the leader. In leader, follower, LF- games, that are repeated many times (50 times in the present game), the cost bias may with time appear to be too large, and the leader—follower sequence changes, possibly with a concomitant change in the player’s preferences. See discussions in e.g., Shachat et al. [1], Nie [2] and Nie [3].

The question addressed in this paper is: How does the player’s observation of each other’s investments, moves, and temporary and accumulated payoffs affect their roles as leaders or followers? A related question: will the role of leadership always produce the highest payoff? The game we discuss is an unprofitable game that shows several traits common with trust games. Below, we discuss possible real world realizations of the game. In a subsection to the discussion section we show results from other applications of the LF- method that we belive support its application to repeated games.

We present a simple method, the leader -follower, LF- strength method, for identifying leaders and followers in repeated games. In experimental games we observe the actions, but generally not the strategies that generate them, e.g.,Martin et al. [4]. However, the LF- method can also be used to identify information that the players use developing their strategy, assuming that i) the information is associated with changes in the strategy and ii) it comes before, that is, it is a leading variable to strategy changes. Other methods for inferring strategies from observations are given in Engle-Warnick and Slonim [5] and Dreber et al.[6]. We believe the assumptions required for using the method will apply to most repeated games, in particular to asymmetric games where the players contest in different roles.

One may visualize the “moves” by a leading player, A, as a time series that shows a peak (or through) before the peak (or through) of a “move” time series of a follower player, B. We assume that the moves of the leading player A, are used as part of the information that player B uses to decide on her or his moves.

The method allows us to determine, for moving triplet of rounds between two players, who is leading the game. The technique distinguishes itself from other methods for establishing leading–lagging characteristics in that it can be applied, although not significantly, to very short time series, n ≥ 3. By using the method break points for changes in leader–follower pattern strategies can be identified as well as short term anomalies in generally persistent leader- follower sequences.

The present method is to our knowledge for the first time applied to game theory. Since the method only requires short time series, say, 10 rounds, it is also applicable to actual situations. However, we demonstrate the method for identifying leader- follower relationships by applying it to a two-player, two- action, asymmetric, unprofitable game with unique mixed strategy equilibrium. An unprofitable game is one in which the minimax and the Nash equilibrium solutions are distinct, but yields the same expected payoff for each player. The game was designed and explored by Noussair and Willinger [7], hereafter N&W. In this type of games the players may be able to get close to the efficient outcome by “alternating” their moves successfully. In contrast, in prisoner’s dilemma games there may be cooperative equilibria. The game is useful for studies of leaders and followers, because both players have incentives to build trust by stepwise strategies that would be reflected in a leader–follower sequence.

### Trust and its objectives

In repeated games, trust is an important variable [5,8,9]. The present game, although not being a trust game under standard assumptions, invokes many of the traditional dilemmas in such games, i.e., how to establish reciprocal trust and how to perceive the objectives of the game. The objective for extending trust depends upon how the players see the game, e.g., if the play is perceived as a rank-order game (the objective is to earn the most) or as a pay—for–performance game (the final outcome counts and trust becomes a means to achieve the goal), Fudenberg et al. [10]. The players can of course see the game differently. Given several assumptions, rank-order goals give stronger motivations than pay-for performance goals, that is, being winner rather than looser gives a greater motivation than a high absolute output [11]. Inequality aversion would contrast with rank–order games, and pure altruism would contrast with pay-for performance goals.

Trust depends on the personality of the contestants, but also on generic attributes, like social distance, socio-economy, gender and race, and on the perceived duration of the game. Engle-Warnick and Slonim [5] found that in a repeated trustor and trustee strategic game trust fell and strategies changed rapidly in a finite game, but not in an “infinite” game.

### Possible real world applications

The asymmetric game we study could be regarded as a first step towards investigating the periodical corporate profit sharing between investors (e.g., to secure continued investments) and corporate managers (e.g., to develop the firm through research and development), although “the real world game” has several attributes not included here, see for example Nocera [12]. Other possible applications for the present method would be repeated games where the player tries to develop a reputation for a certain kind of play. As an example of real repeated games, Fudenberg and Tirole [13] offer the central bank that implements the monetary policy it announces as a leader–follower game. From the monetary policy announced and the economy’s reactions, our method should be able to identify if “the economy “follows the central bank’s announcements or vice versa. Fudenberg et al. [10], found that more players chose Cooperation in the first round than during subsequent rounds, and Au and Komorita [14] found that an initial cooperative strategy strengthened further cooperation. By identifying changing points our method could be used to identify exogenous factors that change leader and follower roles.

The study makes four contributions to games where leading and following is an issue. i) *Payoff variables*. We found that Row player (metaphorically, the investor) most often leads the game in its initial phase, and that the payoff for both players was highest if Row was the leader of the game. However, Column player (the manager) consistently obtained the highest payoff. ii) The game has an efficient alternating “give” and “take”–strategy, but this strategy is either not identified or not accepted during the 50 rounds. In a decision making context, the result suggests that sequential “give” and “take” solutions may be hard to achieve. iii) *Information that forms strategies*. During 50 rounds, leaderships switched on the average 5 times between the players. We found that the most important information for the player’s actions was Row’s investment (and moves, they were similar), and thereafter Column’s payoff. Aggregate payoffs did not appear to be important. iv) *The method* we use to identify leaders and followers is new to game theory, and should be very useful for analyzing repeated games that involve building of reputation and trust. By applying the method to practical games, it should be possible to identify the type of information the players actually use.

The rest of the paper is structured as follows. The experimental design is presented in the section below and give an overview of possible strategies the players can chose. The section ends with four hypotheses. The next section presents the methods used to analyze the results of the game. We thereafter show the results obtained by the new method and discusses the result in the last section.

## Experimental Design

We analyze subject pairs 1 to 8 from N&W. Subjects were selected by a random draw from 1500 volunteer students from various disciplines in three different universities in Strasbourg, France, and they were unknown to each other from the start of the game. The normal form of the game is shown in Table 1. The game is played for 50 rounds and the players knew this limit at the beginning of the game. The experimental currency units, ECU, that were converted to French Franc (about 0.1 Euro) at the rate of 1 Euro = 200 ECU. We use the terms Row and Column player and Up (U) and Down (D) and Left (L) and Right (R) for the moves. (The original study uses also other terms, see S1 Excel. Data and calculations) spread sheet. We use the term “society” (S) for the sum of Row’s and Column’s payoffs. In the present game with two players, the Row player shifts the outcome between small, 0 or 10, or large, 190, payoffs for both players, whereas Column player determines who gets a non-zero payoff. Thus, the players can both extend and receive trust. The game is played for 50 rounds, suggesting that the contestants establish a concern for future relations, and that they can change strategies several times during the game.

**Table 1:** Normal form for the game studied.

		Column Player (2, B)	
		**L** (100)	**R** (0)
Row player (1, A)	**U** (100)	190, 0	0, 190
	**D** (0)	0, 10	10, 0

Numbers in parentheses show # tokens that would cause Row and Column players’ moves to be determined with certainty. Numbers between, but not including, 0 and 100, give increasing probability for move up for Row and move Left for Column. The alternative nomenclature in parentheses refer to the original article by *Noussair and Willinger [7]*.

Both players receive information that can help them update their beliefs about their opponent’s type, e.g., whether the contestant follows a win–lose strategy or a high payoff strategy. Thus, the game is played under an information disclosure regime.

Both players first choose their strategy and then invest an amount of tokens between 0 and 100 on the strategy chosen. Based on the number of tokens, an exogenous random device choses U or D for Row player and L or R for Column player. The relative probability for choosing U vs. D or L vs. R is determined by the proportion of tokens assigned to each alternative. Assigning 100 tokens to one choice would cause that choice to be carried out with certainty. We use the term “invest” for the number of tokens placed on a move, and “move” for the actual resulting moves: U or D, L or R. The number of tokens only determines the probability for choosing a move, but has no direct impact on the earnings.

A timeline for the play would be as shown below, i) Row and Column, R and C, make decisions, **D**, simultaneously; ii) depending upon the number of tokens placed on each of the two alternatives, a probability p (corresponding to #tokens) is determined by a randomizing device for choosing a move; M; iii) depending upon the moves, each player obtains a payoff according to the payoff matrix in Table 1. At each decision point **D**_R_ and **D**_C_ information to the left of the time axis is available to the players.

DR(#tokensonUandD;U+D=100)→MR(U(p),D(1−p))→earningsPR,PC,ΣPR→DR(nextround)

DC(#tokensonLandR;L+R=100)→MC(L(p),R(1−p))→earningsPC,PR,ΣPC→DC(nextround)

The game results in 5 time series for each player, Table 2. Since all series show quasi cycles it is reasonable to assume that they may also reflect changes in the player’s deliberations and strategies that last for several rounds.

**Table 2:** The game is a two by two unprofitable, asymmetric game.

	Row (investor)					Column (manager)				
Round	Inv	Move	Pay	CPa	DeCP	Inv	Move	Pay	CPa	DeCP
**1**	60	Up	0	0	107,7	0	Right	190	190	131,6
**2**	100	Up	190	190	-46,4	100	Left	0	190	202,2
**..**										
**50**	100	Up	0	1610	-92,6	0	Right	190	4830	-182,0

The table shows the first two and the last round for pair 2 in session 1. “Round” is round number, Row is called “investor” and Column is called “manager” because of their roles in the game. “Inv” is investment in “tokens”. For **Row** 0 = Down, 100 = Up, increasing number of tokens > 0 gives increasing probability of Up. For **Column**: 0 = Right, 100 = Left, increasing number of tokens > 0 gives increasing probability of Left. “Move” is the actual move. “Pay” is payoff. “CPa” is the cumulative payoff and “DeCP” is the detrended cumulative payoff. It is the residual after subtracting a 2^nd^ order polynomial function fitted to the cumulative payoff against rounds. The investments column is used for the analysis of leaders and followers, the other columns are used to investigate what type of information the players use in deciding on their next investment.

### Candidate strategies

We have identified seven candidate strategies for the game, but several others could also be listed: i) a strategy which is named cooperative solution (CO) is for Row always to play U and for Column to play L with probability 0.5. The expected outcome for both is 95 ×50 = 4750. To play this strategy the players have to extend trust to each other and they will both likely adopt a pay-for-performance approach. ii) Random play gives 50 × 50 = 2500 for both players. It could result if the players do not decode the game. iii) There is a mixed strategy equilibrium, MSE, that gives 9.5 × 50 = 475 for each player. iv) An altruistic strategy could give the game’s maximum payoff, 190 × 50 = 9500, for one of the players. Then either the Column player is an altruist, or the players have made deals that are not allowed within the rules of the game. v) A minimum payoff solution would occur if Row always plays D and Column always plays either L or R. Since Row will know that Column will decide who gets the payoff, she can decide to let Column win, but with the least payoff possible. There is no trust, the Row player is probably a rank-order player seeing that she must give in, but also seeing that she can punish her contestant. vi) A “best response updating rule” as well as “Win-stay Lose- shift” rule will give a significant leader role for Column player and a significant follower role for Row player, although these terms give no meaning for games with fixed strategies. However, these games presume that none of the players search for a higher total payoff. vii) The last strategy includes an initial search and recognize phase that may end up in a cooperative or collusion strategy. For this strategy the players have to signal intent and honesty, and must recognize signals from the other player. The strategies i) to vi) will only give non-significant leader- follower signatures or the significant signatures are trivial. The last strategy may give significant and interesting LF- signatures.

The game is a potential leader-follower game because the Row player has the possibility to determine if the game’s payoff will be 190 or 10. Following Lunawat [15] we use the term “investor” for this player. Other terms for players with similar roles are “trustors” and “trustee”, e.g., Engle-Warnick and Slonim [5]. Column determines who should receive the non-zero payoff, and we use the term “manager” for this player. Both terms also imply attributes that are not implemented here.

### Previous results

Noussair and Willinger [7] discuss the results of the present non profitable game played by 8 pairs and show that the average choice is 45 tokens for the Row players on U and 28.3 tokens for the Column players on L. Furthermore, their results show that 75% of the Row players and 45% of the Column players use explicit randomizing devices. See S1 Excel. Data and calculations.

### Hypotheses

Firstly, we hypothesizes that at the beginning of the game Row player will be leading the game and Column player will adapt to Row players choices. We hypothesize also that Row player will have accumulated a higher payoff than Column player at the end of the game, because the Column player have to encourage, or play tribute to, Row player to invest high, e.g., Hermalin [16] on paying tribute. *Secondly*, most of the payoff will occur during the last half of the game because optimal strategy choices are then presumably well known. *Thirdly*, we hypothesized that the players switch between leader–follower strategy depending upon their own aggregate payoff, and they would switch frequently allowing the players to communicate efficiently, giving Row incentives to choose high payoff. Alternatively, the players would smooth the information they obtain, and thus postpone switching until trends become stable (e.g., St.Dev (x) << x, n > 3). The rationale for the smoothing procedure is that economists tend to smooth time series, like inflation series, Woodford [17], when they respond to highly volatile information, and we believe that the players in this game behave similarly. *Fourthly*, we hypothesizes that the LF—method used to identify leaders and followers will not result in trivial leader-follower signatures, even though the “best” solution, the cooperative game, CO, would result in such a signature. The rationale is that in all games the players will use time and rounds to internalize the rules and to assess what type of contestant they are playing with. King-Casas et al. [9] suggest that players use 14 seconds to establish “intention to trust”. However, this is a much shorter time span than we would assume relevant for the N&W game. Fudenberg et al. [10] suggest that in their prisoner’s dilemma game even eight rounds (range 1–15), are not sufficient to learn the optimal response to the game.

## Method

The data for the present study were obtained from a previous publication by Marc Willinger, University of Montpellier, Montpellier, France. In the present study we use four major methods in addition to normal regression analysis. The first method: the leader -follower, “LF- strength method” is used for detecting “Who follows who?” and is also used to examine whether signals for changing strategy come before the strategy is actually changed. The second method is principal component analysis, PCA, but we only use it to identify candidate regressors for multiple linear equations. The third method is the smoothing algorithm. Lastly, we outline a method for identifying significance levels.

### The Leader Follower- method

**Fig 1:**
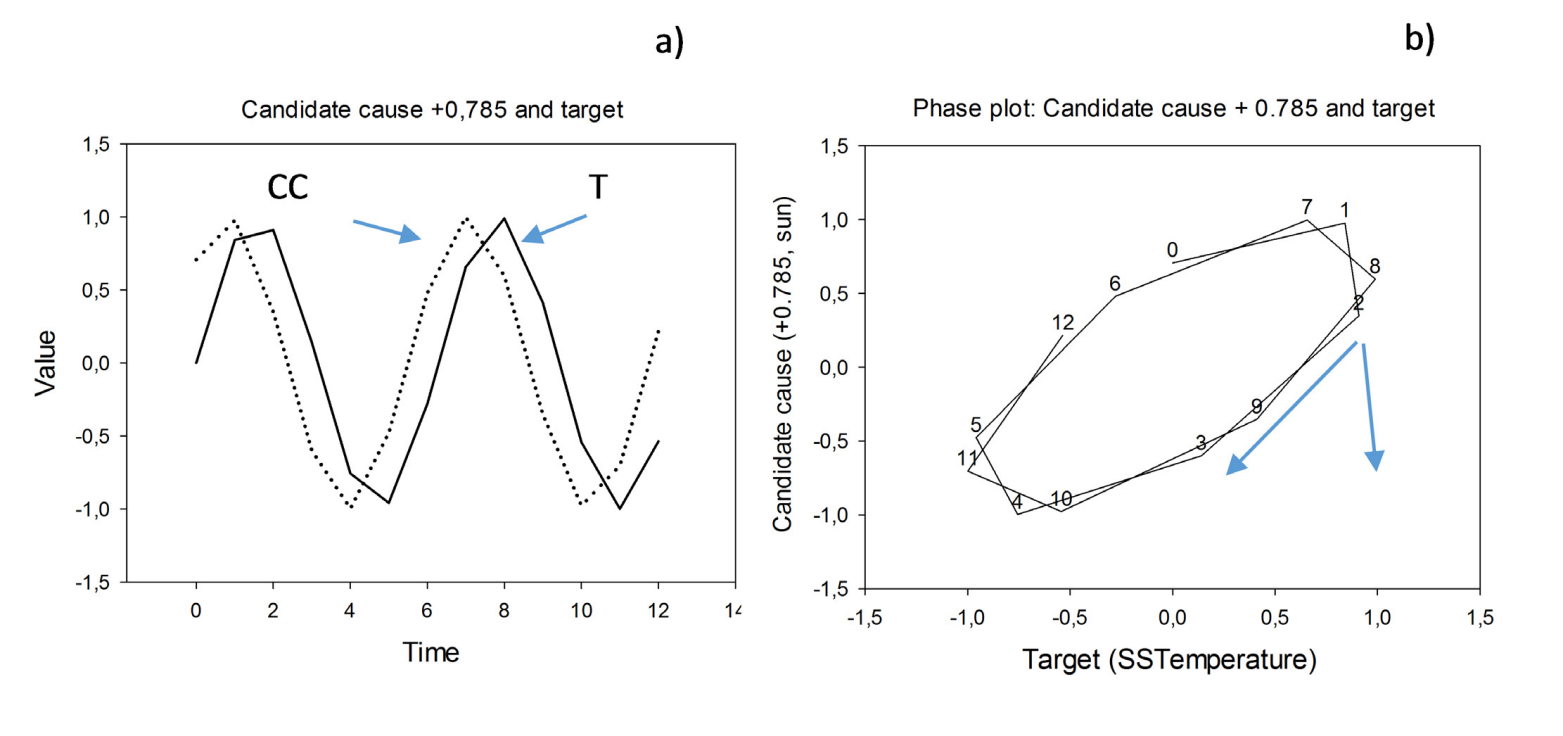
**Time series (left) and phase plots (right)** a) Two sine functions: CC is candidate cause and T is target. The candidate cause, CC, peaks before the target, T. b) In a phase plot with T on the x- axis and CC on the y-axis the time series rotates clock-wise (negative by definition), θ is the angle between two consecutive trajectories.

The basis of the method is the dual representation of paired cyclic time series, x (t) and y (t), as time representation (the x- axis represents time) and as phase plot where the paired time series are depicted on the x-axis and the y-axis on a 2D graph. If one series leads another with less than ½ a cycle length (for example by having a causal effect on the other), then we will have persistent rotational direction of the series trajectories in the phase plot. Fig 1A and 1B give an example with x (t) = sin t and y (t) = sin (t + 0.785). To choose a well-known example of leading and lagging variables, let the first series, x(t), represent sea surface temperature, SST, normally peaking in July–August on the western hemisphere, denoted T in the graph. The second series, y(t), could represent Sun insolation peaking in June, denoted CC in the graph. Since Sun insolation is associated with heat transfer to the sea surface, CC is a candidate cause for T. Thus, CC should peak before T, as it does in the figure. Real pairs of Sun insolation and SST do the same [18]. A detailed explanation of the method is given in Seip and Grøn [19] and example calculations are shown in S1 Excel. Data and calculations.

A basic assumption for this study is that a player represented with a series that leads another is also a leader and the other player a follower. The method consists of 5 steps and is explained with reference to an example with data from the game studied: We use pair no 2 with players 3 and 4 because they were the pairs with highest payoff in the study (N&W 2012).

#### Step 1

**Fig 2:**
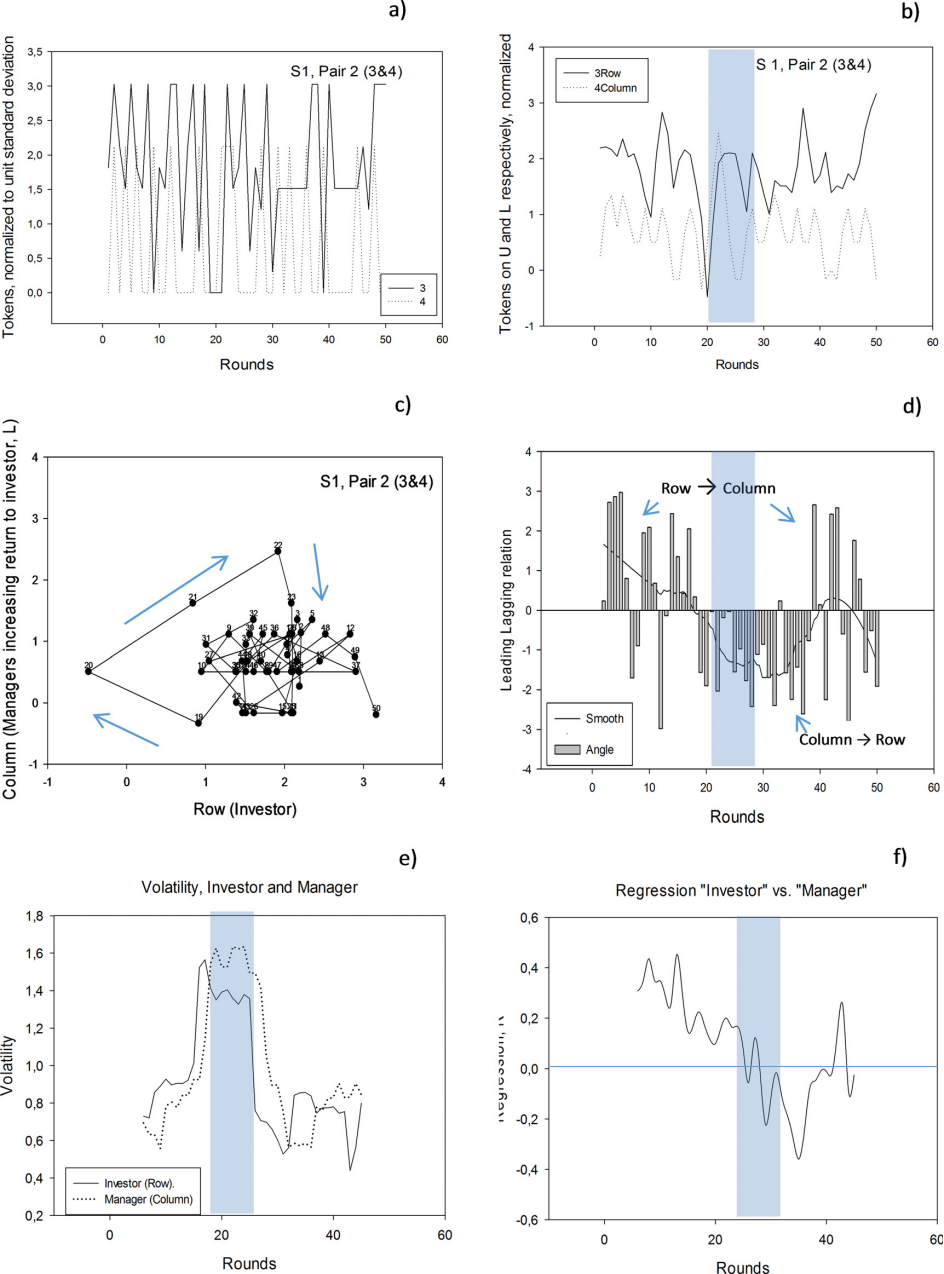
Graphical presentation of Column and Row players’ investment; Session 1, Pair 2. a) “3” is Row player, “4” is Column player. A high value means a high value on Up for Row (high investment) and a high value on Left for Column (Row gets the non-zero payoff). All values have been standardized to unit standard deviation. Shaded areas emphasize rounds 18 to 24 that are discussed further in the text. b) Slightly smoothed versions of the graphs in “a”. c) Phase plot for the time series. Numbers designate round numbers. If trajectories rotate clock-wise (negative per definition) as arrows indicate for rounds 18 to 24, the Column player (the y-axis variable) leads the Row player (the x-axis variable). The rotational pattern corresponds to the two left panels in Fig 1. Note that the angles are measured as angles between compass directions, the angle between points 20–21 and 21–22 is small. d) Who follows who? Row player and Column player. The actual angles of rotation (bars) and smoothed (line). e) Running average volatility (n = 10) as standard deviation for the two series in b. f) Running regression coefficient, R for regression between the two series in b. See text.

We normalize the data to unit standard deviation, e.g., as in Fig 2A for investments. In this way, the time series have similar range for the observations. In this step we also smooth the series to avoid singularities in the subsequent calculations. With smoothing we also see trends in the data more clearly, Fig 2B. (See smoothing section below.)

#### Step 2

We plot pairs of series as phase plot, that is, a plot with values for one series on the x-axis and the simultaneous value for the other series on the y-axis. If the two series show similar, but cyclic patterns shifted in time, then a clock-wise rotation in the x-y-diagram (the phase plot) shows that the y-axis variable leads the x-axis variable. In the present study we plot the Row player’s investment on the x-axis and Column player’s investment on the y-axis, Fig 2C. The clock-wise rotation suggested by the arrows in the figure shows that for the rounds 18 to 24 Column peaks before Row, and then Column is a leader for Row. (Fig 2B; it is more difficult to see the pattern in the rest of the series.)

#### Step 3

We give the Leading lagging relationship a numerical representation: *LL- relations*. To see which variable that peaks first, we quantify rotational directions and apply a strength measure that expresses the persistence of one rotational direction for the trajectories in the phase plot for the paired time series. The formula for quantifying the rotational direction, θ, is,

$$\theta =sign\left({\bar{v}}_{1}\times {\bar{v}}_{2}\right)Acos\left[\frac{{\bar{v}}_{1}\;{\bar{v}}_{2}}{\left|{\bar{v}}_{1}\right|\;\left|{\bar{v}}_{2}\right|}\right]$$ (1)

Implemented calculation of Eq (1) is available from the author.

#### Step 4

*The strength*, LL—strength, of the mechanisms that cause two variables to either rotate clock-wise or counter clock-wise in a phase portrait, is measured by the number of positive rotations (counter clock-wise rotations by convention) minus the number of negative rotations, relative to the total number of rotations over a certain period, in this study, 9 rounds,

LL=(Npos−Nneg)/(Npos+Nneg). (2)

We use the nomenclature: LL(x, y) = [–1, 1] for leading- lagging strength: LL (x, y) < 0 implies that y leads x, y→x; LL(x, y) > 0 implies that x leads y, x→y. The LL- strength for the series in Fig 2A is LL = -1.Thus, we can use the rotational directions in phase plots for two cyclic series to infer which series is preceding the other in the sense that its peak (through) is less than ½ of a cycle time before the peak (through) of the other. A good graphical example is shown on Wikipedia [20].

The measured LF- strength captures two aspects of the LF- relationship between paired variables. It obtains a high / low value when the player is consistently leading or following the contestant. However, to obtain a high / low value the two leader- follower series have to change cycle lengths in concert.

#### Step 5

We design a graphical presentation of the results in terms of rotational angles between trajectories in the phase plot, Fig 2D. The graph should be interpreted as follows: the x-axes represents the rounds of play (1 to 50). The y-axis designates “who follows who”. If the y-axis shows positive numbers, the Row player (on x-axis of the phase plot) is leading the Column player (on y-axis of the phase plot). From the example in Fig 2D it is seen that the Row player leads the 20 first rounds, then the Column player takes over for about 20 rounds, and for the last 10 rounds there is no persistent leader–follower sequence.

We report on the volatility of the two series measured by their running standard deviation. Running average (n = 10) volatilities and regression coefficients for the time series in Fig 2B are shown in Fig 2E and 2F respectively.

The leader–follower characteristics of the 8 games played was represented with 8 phase plots (not shown). We quantify the leading lagging relationships and compare the LL- characteristics to the accumulated payoffs that are obtained during the 50 rounds of play. The quantification results in a table with 9 rows and 8 columns with numerical values, one column for each of the 8 games.

### Smoothing

Since the players had the option of choosing 0 and 100 tokens, the paired time series could show series with several 0 or 100 following each other. We therefore smoothed the series slightly to avoid singularities in the calculations, but also to emphasis trends in the games played. The smoothed series determine the portions of the time axis where there are persistent LF–relations, but the series pass through non-significant portions when they cross the time line, that is, when leader–follower roles change. We smoothed the series with the LOESS algorithm, SigmaPlot, using a fraction of 0.1 of the series and a 2^nd^ order polynomial function for interpolation. To examine the type of information players use in making their decisions, the series in Table 2 were smoothed to increasing degree.

### Estimation of significance

We have estimated the significance of the LF-strength measures by calculating 95% confidence estimates for paired uniformly random series with 50 entries, corresponding to the 50 rounds of the games. We repeated the calculations 10, 20, 40, 80 and 160 times (number of games were 8) and found as the asymptotic value for the confidence interval around 1/n ∑ θ = 0.0 [-0.03,0.03]. For series 10 rounds long, the LF- confidence interval was greater [-0.3; +0.3]. We used these last values as a conservative estimate of a practical confidence interval.

### Information retrieval by the players

To identify the information that is used by the players when they decide to change strategy in the leader–follower context, we compared the information variables to the leader–follower sequence expressed by the angles values, θ _i_, i = 2, 49 for the 50 rounds (we use 3 observations to calculate θ, so the first and the last observation do not allow calculation of θ). Since the aggregated payoff variable increases, it was detrended by subtracting a 2^nd^ order polynomial regression of the payoff against time.

We use the series that expresses the leader–follower sequence as consequence, and the information available to the players as possible causal information. To identify the information that probably is used most, we suggest two conjectures that we believe support the information as important for the players’ choices: i) the information is available before the leader–follower decision is made, and ii) there is a close association between the information and the curve that describes the leader–follower sequence. As candidate information series we used the raw series and the detrended accumulated payoff, and series smoothed to an increasing degree from raw to the maximum smoothing (LOESS with moving window of ¼ of time series length and 2^nd^ order polynomial function for interpolation.)

## Results

First we present our results on leader and follower relationship between the Row and Column players. Secondly, we present the results for the game’s payoff as it relates to leader–follower characteristics of the games. Thirdly, we report on the type of information that prompts players to shift strategy.

### Payoff as a function of leadership

We first examine if payoff can be predicted from generic characteristics of the game as it is actually played. The first three rows in Table 3 show the social payoff for the game, and the payoff for each player. Column’s payoff is always larger than Row’s payoff. The next rows, 4 to 9, show characteristics of the 8 games. The 4^th^ row shows the fraction of times Column is leading the game, CL. (Row on x-axis, Column on y-axis). The 5^th^ row shows the number of times the players change in being leading during all 50 rounds, Ch. The 6^th^ row shows the number of rounds Row leads Column at the beginning of the play, RS. The 7^th^ row shows the number of rounds that Column leads. The 8^th^ row shows how many rounds Column leads after Row has ceased to lead, CC (we count the number of times Column is leading the game after Row ceases to lead). The 9^th^ row shows how many rounds Row leads at the end of the game, RE. The 10^th^ row shows the running average (n = 10) regression coefficient for Row and Column’s investments and the 11^th^ row shows the main functional form for the volatilities in Row’s investment. The 12^th^ and the 13^th^ rows show Row’s and Column’s payoff when 50% of the rounds have been played. We screen the result with principal component analysis, PCA, using the parameters as columns and the pairs 1 to 8 as rows in a 9 × 8 matrix.

**Table 3:** Analyzing leader-follower data.

Parameter	Pairs								
	1	2	3	4	5	6	7	8	average
**1.Social payoff; SP**	5720	6440	4820	3380	3560	5000	3380	8060	5045
**2.Row payoff; RP**	1320	1610	1300	1540	1720	1330	640	2910	1546
**3.Column payoff, CP**	4400	4830	3520	1840	1840	3670	2740	5150	3499
**4. Column Leading; CL; %**	0.27	0.41	0.38	0.42	0.35	0.31	0.56	0.27	0.37
**5.Changes; Ch**	5	5	4	7	6	4	5	5	5.1
**6. Row Start. RS; rounds**	0	18	19	1	0	10	21	5	9.3
**7.Col Start; CS; rounds**	0	0	20	9	1	0	0	0	3.8
**8. Column Continue. CC; rounds**	8	22	21	20	10	37	11	17	18.2
**9. Row Ends. RE; rounds**	15	5	10	30	0	3	0	0	7.9
**10 Regresjon, R**	-0,80	-0,73	0,52	-0,86	-0,06	-0,12	-0,83	-0,23	-0.39
**11 Volatilitet**	U shape	Inv U	Inv U	Inv U	Inv U	Inv U	Inv U	Inv U	Inv U
**12. Row payoff, first 1/2**	1020	1000	460	1060	680	830	310	1760	890
**13.Column payoff first 1/2**	2100	1940	1940	980	1000	1930	1730	1540	1645

Rows 1–3 shows payoff; Rows 3–9 characterize leading lagging relationships during the 50 rounds; row 10 shows the trend in running average (n = 10) regression coefficient, R, between the two player investments and row 11 shows curve form describing the running average (n = 10) for the volatility in investments. Rows 12 and 13 show payoff results after half played game. See text for further details.

#### Result 1

**Payoff predictions.** Of the five candidate variables that potentially could explain a high payoff for the players, only two were significant. *Column payoff*, CP, is best predicted by two variables. The best predictor are the number of rounds that Row (investor) is leading the game (p = 0.035) and that Row is starting the game (p = 0.062). Note that C_Leads_ in the equation below is preceded with a negative sign which is equivalent to Row Leading the game.

Cpayoff=7262−13043CLeads+116RStart,R=0.80,p=0.067,n=8,(pCL=0.034.pRS=0.062) (3)

*Row payoff*, RP, is best predicted by the number of rounds that Row (Investor) is leading the game, but not significantly at the 0.05 level. Again, C_Leads_ is preceded by a negative sign.

Rpayoff=3115−4233CLeads,R=0.64,p=0.089,n=8 (4)

There were no significant predictors for Social gain (p > 0.1).

It is interesting that Column (the Manager) obtained the largest accumulated payoff in all games. We examined if there was a relationship between Row’s payoff and the ratio between Row’s and Column’s payoffs, R/C, but found none (p > 0.3).

**Fig 3:**
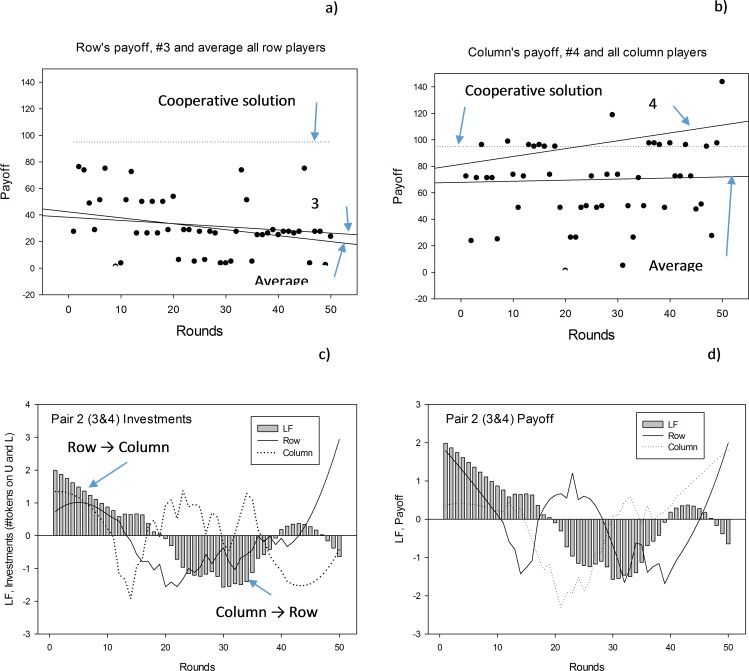
Payoff as a function of rounds. a) Payoff for Row; average all players and player #3. The average cooperative solution is the dashed line (symbols only for the average solution). Average R = -0.45 Rounds + 42.33, r = 0.30, p = 0.037. b) Payoff for Column; average all players and player 4. The dashed line is the average cooperative solution (symbols only for the average solution). Average C = 0.08 Rounds + 67.88, r = 0.04, p = 0.88. c) Leader- follower, LF-relationship (bars) compared with investment (#tokens normalized to unit standard deviation). Positive investment means Up for the Row and Left for the Column player. d) LF- relationship (bars) with Row’s and Column’s payoff.

The Row player received less than half its payoff during the last 25 rounds whereas the Column player received about equal payoff in the first half and the last half of the game, Fig 3A and 3B. Although not significant (p = 0.067), the longer Row (Investor) leads the game in the first half of the game, the larger is Column’s (managers) payoff relative to Row’s payoff.

#### Result 2

**Actual payoff compared to the cooperative sequential payoff strategy, CO.** Generally, the two players did not reach the payoff that corresponds to the efficient cooperative alternating “give” and “take” strategy, Fig 3A (Row’s payoff) and Fig 3B (Column’s payoff). Row was furthest from the cooperative strategy.

### Player’s communication and information use

Let us examine what type of information the players use in making their decision to lead or follow. Since the leader–follower sequence is a function of both players’ moves; a changing point is unknown to both players at the time it occurs. We first make a screening with PCA. Thereafter we show results for pair # 2 as an example, and lastly we make calculations for all 8 games played.

#### Screening

We constructed a PCA matrix for the leader–follower, LF—relationships (as the angles θ_i_ that expresses the LF- relationship) and all information available to the players, that is, their own and their contestants investment and subsequent moves (moves were coded as U = 2, D = 1, R = - 2 and L = - 1) as well as single round payoffs and raw and detrended accumulated payoffs. This was done for all 8 games separately.

#### Result 3

**Players information.** We found that Row’s investment (and moves, they were very similar) and Column’s payoff were the most likely information sources for the choice of being leader or follower. If we assume that the players smooth the information they obtain, the result was strengthened. No other information was close two these two variables in being associated with the LF–curve. To better get an understanding of our quantitative calculations, we first present Game # 2 as an example in Fig 3C and 3D and then present the results for all 8 games.

With references to Fig 3C, the results for investments as independent variables are shown as Eq (5):

LF−relation=−3.58+0.51RowInvU+0.09ColumnInvL,r=0.50,p=0.001,(pRow<0.001,pColumn=0.48) (5)

Row’s investment is the strongest information carrier (β–coefficient = 0. 51, p = 0.001), whereas Column’s payoff contributes less, and non-significantly, (β–coefficient = 0.09, p = 0.48).

With reference to Fig 3D, the results for payoff as independent variable are shown as Eq (6):

LF−relation=−1.333+0.303CPayoff,R=0.30,p=0.019 (6)

In terms of payoff, only Column’s payoff affects the LF—relation, but with less explanatory power than Row’s investment.

Column’s payoff is closely related to Row’s investment on U:

CPayoff=−0.0+0.82RowInv,R=0.82,p<0.001,n=50 (7)

We found no relationship between payoff and the number of switchings between leader and follower roles, p > 0.3. The number of switchings was relatively constant. Also, the average investment from Column that would give Row a non-zero payoff was 28.3%, much less than the 50% that could have given an equal payoff to the two players.

Firstly, we examined with simple regression whether investment and payoff were correlated with the LF- curve that expresses the leader–follower sequence for all 8 games. Secondly, we examined, by using the LF- strength method, whether investment and payoff precede the LF—curve. In half of the games investment and payoff correlated, and preceded, the LF–curve. Smoothing Row’s and Column’s investment and payoff gave generally higher correlation and more significant leading signatures (in 80% of the games. Smooth curves are not shown, but correspond to visual smoothing of the curves in Fig 3C and 3D)

#### Result 4

**The Leader–follower method.** The LF—method gave clear signatures for leader–follower sequences in all 8 games. In no game were the results trivial. “Trivial” meaning that there is a single leader during all 50 rounds, one or both of the players play stochastically all the time, or the leader and follower use a fixed strategy for the whole game. The frequency with which the players changed their investment showed that in 7 of the 8 games both players change little at the beginning and the end of the game, but much in the middle of the game (inverted U form for volatility).

## Discussion

In this section we first examine the result of the N&W game in terms of payoffs and information retrieval. Lastly, we discuss the method. We use the term “Investor” for the Row player and the term “Manager” for the Column player to make it easier to remember the roles of the two players.

### Payoff

The game can metaphorically be compared to the investor/ manager “game” played when corporate profit is shared, although the game only describes a small part of the mechanisms involved. We found that the longer the investor (Row) is the leader of the game, the higher is the payoff for both investor (Row) and manager (Column). However, it is always the manager that achieves the highest payoff. Thus, whereas the investor is most commonly the leader of the game and is the player that secures a high common payoff, the investor is always beaten in obtaining the highest payoff. The results support our first hypothesis, that the Investor has a decisive role in obtaining high payoff for both players, but contrasts with the hypothesis in that the Manager, not the Investor, benefited most from the game. The result is consistent with findings by Lunawat [15] in an investment/ trust game that the manager returns less than 50% to the investor, and with Dreber et al. [6] that altruism is not a major issue in (simple) repeated games. It appears that paying tribute to the investor [16] in this type of games is also a non-frequent strategy. The player’s profit did not approach the alternating “give” and “take” strategy, contrasting with our *second* hypothesis. A practical consequence of our results is that coordinating such a strategy may be difficult. We have found no systematic evidences on corporate profit sharing between shareholders and investments in the literature.

A tentative explanation of this result is that Investors are eager to achieve high payoff (play Up, a “lottery—mindset”) and react slowly to the managers choices of keeping the high investment for herself. (She plays persistently Right). The N&W game appears to be more like a “pay-for–performance” game than a “rank-order game”, but the signatures are not clear. The highest total payoff during one game was 5150 for the Manager, that is, only 54% of the maximum possible payoff of 9500.

Fig 2E showed that the players tend to be more volatile and change their strategy faster during the middle of the games, (volatility has an inverted U-shape for all games except game #3.) From Fig 2F is seen that the regression coefficient, R, between investors and managers time series is first high, indicating that the players follow each other closely. Then the distance between the cycles increases and R becomes smaller indicating either that there is a LF—relationship between the investor and manager (assuming that cycles are present), or that both behave stochastically. Significant negative values of R suggest that cycles are counter-cyclic. The negative trend for R with the number of rounds apply to all games except game #3, Table 3, Row 10.

### Information retrieval and use

Our third hypothesis was not supported; the players did not examine their accumulated or detrended accumulated payoff to determine if they should lead the game. Instead our results showed that for all pairs, the most important contribution to the choice of the player’s leader–follower strategy was the investment the Investor makes. On a second place comes the Manager’s payoff.

We offered two conjectures for information retrieval, i.e., information is available before decisions are made and information and decisions are closely associated. Both appeared to be supported, in the sense that we found variables that satisfied both conjectures, and the information carried by the variables were reasonable candidates for the players’ decision making.

Our fourth hypothesis was that the contestants would frequently switch roles as leader and follower, allowing the players to efficiently communicate trust and honesty. However, there was no relation between the frequency of switching leadership and payoff. Furthermore, we would have anticipated high volatility in investments at the beginning of the games, allowing retrieval of information on the contestant’s type and character, but most games (7 of 8) showed the highest volatility in the middle of the game. We do not yet have a rationale for this behavior.

The N&W repeated game appears not to have given the players sufficiently incentives to reach a social payoff that was close to the efficient solution. The game was played under full disclosure, e.g., as in Lunawat [15] and Martin et al. [4], but there were no exogenous clues that could increase trust in the game. An interesting question is if there is some minimal, practical instrument that would enhance a goal of maximum social, equally distributed, payoff.

### The method

In repeated games, it may be difficult to find “ground truths” that can be used to verify the method. However, “ground truths” exist within other disciplines. In macroeconomics there are “common knowledges”; e.g., some leading indexes are almost universally accepted to be leading the gross domestic product, and has been shown to do that. The method has also shown that corporate profit in the US leads employee’s compensation with about 10 quarters. [21]. In addition, the method has been used to examine leader, follower traits in oceanographic and biological systems [18,19,22]. The method quantifies LF- relationships, but in most cases, inspecting the time series, the same LF- relationships can be inferred. We used the method for two purposes: identifying at which time, and for how long, a player was the leader of a game, and we used it to identify what type of information the two players depended upon for their strategy. Our method should be useful also for many other applications. However, a leading- lagging sequence does not imply causation, but with additional information it may strengthen a potential causal relationship.

We would like to apply the method to a repeated game where leaders, followers, and their information retrieval procedures are known or estimated by other methods than ours. We believe that the new method will allow experimentalists to design the experimental conditions so that they more closely reflect the intended real conditions, but still be able to identify strategies and behavior of interest.

## Conclusion

We have obtained four major results: A novel method for identifying leading–lagging, LF—relations, has been demonstrated by applying it to a repeated game with two players. At present it is best suited for games that do not have too high frequency of binary choices (rather on a scale from 0 to 100 than only 0 and 1), and for games where strategy changes over several rounds (> 10) are of interest. *Secondly*, we found that in an investor–manager type game with 50 rounds, when the investor leads the game, profit is highest for both players, but the manager always gets the largest profit. *Thirdly*, in this game with an efficient alternating strategy requiring sequential profit sharing, the players do not identify or accept this strategy. *Fourthly*, we identify from the course of the game what type of information both players use to decide on their next move.

## Supporting Information

S1 Excel Data and example calculation for Seip. Leader and followers.xlsx.
(XLSX)
